# Size of decompressive craniectomy as prognostic factor in space-occupying ischemic cerebellar stroke –a multicentric retrospective study

**DOI:** 10.1016/j.bas.2025.105911

**Published:** 2025-12-14

**Authors:** Silvia Hernández-Durán, Johannes Walter, Daniel Dubinski, Obada T. Alhalabi, Milos Arsenovic, Daniel Cantre, Nazife Dinc, Judith Dremel, Nima Etminan, Thomas M. Freiman, Kiarash Ferdowssian, Erdem Güresir, Katharina A.M. Hackenberg, Motaz Hamed, Andreas Kramer, Christopher Krämer, Beate Kranawetter, Tim Lampmann, Anne Neumeister, Artem Rafaelian, Florian Ringel, Veit Rohde, Jan Hendrik Schäfer, Michael Schwake, Christian Senft, Alexander Storch, Moritz Thiel, Merih Turgut, Andreas W. Unterberg, Peter Vajkoczy, Hartmut Vatter, Martin Vychopen, Johannes Wach, Matthias Wittstock, Florian Gessler, Sae-Yeon Won

**Affiliations:** a Department of Neurosurgery, University Medical Center Rostock, Rostock, Germany; b Department of Neurosurgery, University Hospital Heidelberg, Heidelberg, Germany and University of Heidelberg, Medical Faculty, Heidelberg, Germany; c Department of Neurosurgery, University Hospital Göttingen, Göttingen, Germany; d Department of Neuroradiology, University Medical Center Rostock, Rostock, Germany; e Department of Neurosurgery, University Hospital Jena, Jena, Germany; f Department of Neurosurgery, University Hospital Mannheim, Mannheim, Germany; g Department of Neurosurgery, Charité – Universitätsmedizin Berlin, corporate member of Freie Universität Berlin, Humboldt-Universität zu Berlin, and Berlin Institute of Health, Berlin, Germany; h Department of Neurosurgery, University Hospital Leipzig, Leipzig, Germany; i Department of Neurosurgery, University Hospital Bonn, Bonn, Germany; j Department of Neurosurgery, University Medical Center of the Johannes Gutenberg University of Mainz, Mainz, Germany; k Goethe University Frankfurt, University Hospital, Department of Neurology, Frankfurt am Main, Germany; l Department of Neurosurgery, University Hospital Münster, Münster, Germany; m Department of Neurology, University Medical Center Rostock, Rostock, Germany

**Keywords:** Cerebellar stroke, Suboccipital craniectomy, Surgery for stroke

## Abstract

**Introduction:**

In cases of space-occupying cerebellar ischemic strokes, guidelines recommend suboccipital decompressive surgery (SDC). While in supratentorial hemispheric stroke, the size of the bone flap has been the subject of many studies and ample debate, no studies have been conducted to determine the optimal size of the bone flap to be removed in SDC.

**Research question:**

To determine the optimal size of SDC in ischemic cerebellar stroke.

**Methods:**

This is a multicentric retrospective study of patients undergoing SDC for ischemic cerebellar stroke. SDC size was determined in two perpendicular planes on early postoperative CT scans: (a) maximal lateral extension (L) and (b) maximal craniocaudal extension (CC) in cm. The primary endpoint was functional outcome according to modified Rankin Scale (mRS) at three months. Secondary outcome was mortality at three months, as well as surgical complications.

**Results:**

A total of 88 patients were included in the final analysis. The mean L diameter of the SDC analyzed was 7 cm (SD 1.5), whereas the mean CC diameter was 4.4 cm (SD .8). When dichotomizing patients based on a threshold of L ≥ 6.5 cm, favorable outcome was more likely in the group with L ≥ 6.5 cm (OR = 3.23, 95%CI 1.02–10.56, p = .045). No statistically significant differences were observed in mortality at three months (OR = .66, 95%CI .24–1.78, p = .40).

**Conclusions:**

In ischemic cerebellar stroke, a suboccipital craniectomy with a maximum lateral diameter of ≥6.5 cm appears to yield better functional outcomes than smaller ones. Prospective studies are needed to confirm these results.

## Highlights

• Largest multicenter study on craniectomy size in cerebellar ischemic stroke.
• Lateral diameter ≥6.5 cm linked to better 3-month functional outcomes.
• Suggests decompression of ∼50 % cerebellar convexity for optimal results.

## 1 Introduction

The life-saving role of decompressive craniectomy (DC) in malignant hemispheric stroke has been established by randomized controlled trials (Vahedi et al., 2007) and this surgical intervention is now an essential part of stroke treatment. Since the first descriptions of DC in 1894 by Annandale (Rossini et al., 2019; Ransohoff and Benjamin, 1971), where a large bone flap was performed from the glabella to the inion anteroposteriorly and to the temporal squama laterally, surgical techniques have evolved, and invasiveness decreased. Thus, several studies have been conducted to determine the optimal size of the bone flap in DC. Current evidence supports that the bone flap should have a minimum diameter of 12–14 cm(Wirtz et al., 1997; Lehrieder et al., 2023): smaller DCs have been associated with a higher risk of transcalvarial herniation and contusions at the bone edges due to shear injury, whereas larger bone flaps of more than 14 cm in diameter have been linked to a higher incidence of bleedings, damage to bridging veins or sinuses, postsurgical hydrocephalus, and sinking skin flap syndrome (Wagner et al., 2001; Kurzbuch, 2015).

In the posterior fossa, guidelines recommend decompressive surgery in cases of space-occupying cerebellar ischemic stroke (SOCS) (Wijdicks et al., 2014; van der et al., 2021). However, surgery in these cases has not been amply studied and European guidelines (van der et al., 2021) emphasize the lack of evidence concerning SOCS and which patients would benefit from surgical intervention. Initial reports on surgery for posterior fossa infarction presupposed a wide exposure of the cerebellum, from the transverse sinus superiorly, to the sigmoid sinus laterally and the foramen magnum caudally (Tandler and Ranzi; Fairburn and Oliver, 1956). Analogous to DC, the technique for suboccipital decompressive craniectomy (SDC) in cerebellar ischemic stroke has changed and become less invasive. However, no studies have been conducted to further describe the surgical nuances of SDC or determine the optimal size of the bone flap to be removed in posterior fossa ischemic stroke. Therefore, in this study, we assessed the association of the dimension of SDC and functional outcome.

## 2 Materials and methods

This study constitutes a subgroup analysis of a retrospective, multi-center study of patients undergoing SDC for the treatment of SOCS between January 2011 and July 2024 at eleven academic centers in Germany. All centers followed a standardized escalation pathway consistent with contemporary American Heart Association/American Stroke Association (AHA/ASA) recommendations for cerebellar infarction, which include close neurological monitoring, optimization of systemic parameters, and early recognition of deterioration. Specifically, all patients received what each institution defines as maximal medical therapy prior to decompression, including head-of-bed elevation, blood pressure optimization, ventilatory support as needed, and ICU-level monitoring (Wijdicks et al., 2014).

Surgical indications at these academic centers were according to current guidelines; decompression was indicated after medical management had failed to prevent neurological deterioration or if the space-occupying effect suggested impending brainstem compression. According to the joint recommendations of the German Union of Anesthesiologists (BDA), the German Union of Surgeons (BDC), the German Union for Surgical Management (VOPM), the Austrian Union for Surgical Management (VOPMÖ) and the Swiss Association for Surgical Management (SFOPM), surgeries once indicated had to be carried out within 1 h of indication (N0 to N1), thus rendering the time from last-well-seen to surgery homogeneous in the cohort (Bauer et al., 2020).

Assessment of infarct characteristics was based on baseline non-contrast CT. Because CT has limited sensitivity for early or subtle ischemia in the brainstem, precise and reliable classification of brainstem involvement was not feasible. Surgical indication incorporated clinical and radiological judgment by stroke and neurosurgical specialists; patients with clear major brainstem involvement or radiological evidence of non-survivable brainstem injury were not considered candidates for decompression in interdisciplinary consensus. Thus, the surgical cohort inherently reflects patients with predominant cerebellar infarction rather than extensive brainstem stroke.

Surgical techniques were subject to institutional standards, thus leaving the concomitant use of external ventricular drains (EVD), C1 laminectomy and duraplasty techniques to surgeon's discretion. Patients in whom intentional necrosectomy or resection of infarcted tissue was performed as part of the primary procedure were excluded to ensure cohort homogeneity. Minor tissue herniation through the decompression site, which is frequently encountered, was not considered as necrosectomy and did not lead to exclusion.The study was carried out in accordance with the 1964 Helsinki Declaration and with approval from the institutional review board (IRB) of the participating centers. Informed consent was not required by the IRB due to the retrospective nature of the analysis.

### 2.1 Patients

We collected sex, age, Glasgow Coma Scale (GCS) at presentation. Relevant comorbidities such as arterial hypertension (AHT), diabetes mellitus (DM), chronic obstructive pulmonary disease (COPD)/asthma, coronary artery disease (CAD), atrial fibrillation (AF), and positive past medical history for stroke were also assessed. Time from ictus to surgery was documented. As per national standards, all patients were monitored and treated in certified interdisciplinary Stroke Units. Postoperative complications, such as pneumonia, urinary tract infections (UTI), surgical site infections (SSI), or cerebrospinal fluid (CSF) leaks/fistulas were also assessed.

### 2.2 Radiographic parameters

**Fig. 1:**
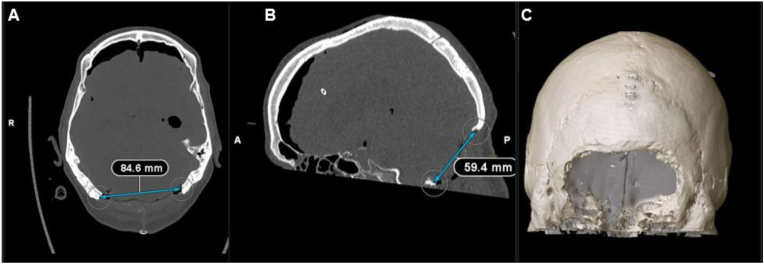
Measurements of craniectomy size in postoperative CT scans. A Determination of maximum lateral diameter on axial CT scan. B 3D reconstruction of postoperative CT scan demonstrating craniectomy size as an ellipse. C Determination of maximum craniocaudal diameter on sagittal CT scan.

The size of the craniectomy was determined in two perpendicular planes on early postoperative CT scans: (a) maximal lateral diameter (L) in the axial reconstructions and (b) maximal craniocaudal diameter (CC) in the sagittal reconstructions, from the superior border of the bone defect to the foramen magnum, in cm (Fig. 1). The area of the craniectomy was calculated assuming that the shape thereof would be an ellipse according to the following equation: $A=\pi *\frac{CC}{2}*\frac{L}{2}$ .The dimensions of the cerebellar convexity were also measured, with the maximal lateral diameter from the right to the left-sided mastoid region.

### 2.3 Primary and secondary endpoints

The primary endpoint of this study was functional outcome at three months, as determined by the modified Rankin Scale (mRS). mRS at follow-up was used in line with prior retrospective studies on malignant infarction. Pre-morbid mRS was not uniformly documented and available history was insufficiently structured to allow reliable imputation; therefore, mRS as functional outcome was presented descriptively. Baseline characteristics—including prior stroke and major comorbidities (hypertension, diabetes, cardiovascular disease, anticoagulation status)—were compared between patients undergoing smaller versus larger decompressions. Functional outcome was dichotomized as favorable for mRS 0–3, and unfavorable for mRS 4–6. Secondary outcome was mortality at three months, as well as surgical complications including SSI and CSF fistulas.

### 2.4 Statistical analysis

Normally distributed continuous variables are reported as medians (and interquartile ranges (IQR)), while frequencies and percentages were used for categorical and ordinal variables. The median values of all the dimensions analyzed were used as thresholds for dichotomization. Then, analyses of N x 2 contingency tables (chi-square test) were performed for the primary and secondary outcomes. Results are reported in odds ratios (OR) and 95 % confidence intervals (CI). Significance was assumed at p < .05.

Statistical analyses were performed with IBM® SPSS® v. 25.

## 3 Results

In total, 117 patients with cerebellar stroke who underwent SDC were identified. Of these, 17 patients (14.5 %) received ample necrosectomy combined with SDC and 12 (10.3 %) did not have full postoperative imaging datasets and/or clinical follow-up at three months, thus precluding an accurate analysis of SDC size in relation to functional outcome. Consequently, 88 patients (75.2 %) were included in the final analysis. Of these, 61 (70 %) received a concomitant EVD as part of their therapy. Baseline characteristics are summarized in Table 1.

**Table 1:** Clinical parameters, interventions, and postoperative course of the entire cohort of 88 patients undergoing suboccipital decompressive craniectomy for space-occupying cerebellar stroke.

Clinical parameters	
Age in years, median (IQR)	62 (56–72)
Male/female, n (%)	64/24 (73/27)
Arterial hypertension, n (%)	63 (72)
Diabetes mellitus type 2, n (%)	30 (34)
COPD/asthma, n (%)	14 (16)
Coronary artery disease, n (%)	9 (10)
Atrial fibrillation, n (%)	19 (22)
Past medical history of stroke, n (%)	8 (9)
Prior use of oral anticoagulants, n (%)	15 (17)
Prior use of antiplatelet agents, n (%)	24 (27)
GCS at admission, median (IQR)	13 (3–15)
Brainstem infarction, n (%)	34 (39)
**Interventions**	
IV thrombolysis, n (%)	27 (31)
Mechanical thrombectomy, n (%)	29 (33)
Concomitant use of external ventricular drain, n (%)	61 (70)
Time to surgery in hours from ictus, median (IQR)	28 (7–52)
**Postoperative course**	
Pneumonia, n (%)	36 (41)
Urinary tract infection, n (%)	5 (5)
Hemorrhagic transformation postoperatively, n (%)	7 (8)
Surgical site infection, n (%)	2 (2)
Cerebrospinal fluid fistula, n (%)	7 (8)
In-house mortality, n (%)	22 (25)

COPD – chronic obstructive pulmonary disease; GCS – Glasgow Coma Scale; NIHSS – National Institute for Health Stroke Scale.

### 3.1 Craniectomy dimensions and outcomes

The median L diameter of the SDC analyzed was 7 cm (IQR 6–7.8 cm); whereas the median CC diameter was 4.5 cm (IQR 3.9–4.9 cm). The median computed area was 25 cm^2^ (IQR 20–30 cm^2^) in the entire cohort. Based on the median value of L = 7 cm, ≥6.5 cm (as the lowest round up value for 7) was used to dichotomize the groups.

**Fig. 2:**
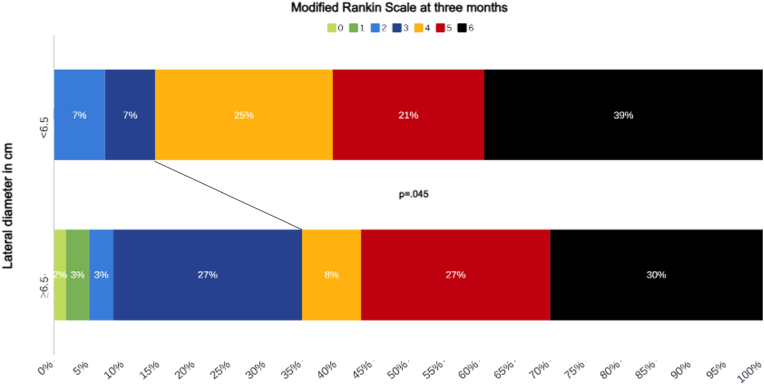
Functional outcome according to the modified Rankin Scale at three months with patients stratified based on the lateral diameter of their suboccipital decompressive craniectomy.

When dichotomizing patients based on a diameter of L ≥ 6.5 cm, favorable outcome was more likely to be achieved in the group ≥6.5 cm or more in L diameter (OR = 3.23, 95%CI 1.02–10.56, p = .045, Fig. 2). The median L diameter of the cerebellar convexity was 14 cm (IQR 13–15). By calculating half of the diameter, which would be 7 cm in our series, around 50 % of the cerebellar convexity could be considered as a sufficient decompression in case of SDC.

In addition, the groups were dichotomized by the median CC diameter of 4.5 cm. Patients with CC ≥ 4.5 cm had 35 % favorable outcome compared to 22 % in patients with CC < 4.5 cm; however, no significance was reached (OR = 1.9, 95%CI = .72–4.81, p = .188). Similarly, when dichotomizing patients according to an area of 25 cm^2^, no statistically significant difference was observed in functional outcome at three months, with 33 % patients with area≥25 cm^2^ having favorable outcome compared to 22 % of patients with area<25 cm^2^ (OR = .57, 95%CI .22–1.48, p = .249). No significant differences were observed in the secondary outcomes, as evinced in Table 2.

**Table 2:** Analysis of secondary outcomes by dichotomizing the group into L6.5 cm and L < 6.5 cm.

	L ≥ 6.5 cm	L < 6.5 cm	OR (95 % CI)	p
Hemorrhagic transformation, n (%)	5 (15)	2 (13)	1.25 (.22–7.27)	.804
Surgical site infections n (%)	2 (1)	0 (0)	2.30 (.10–51.06)	.598
Cerebrospinal fluid fistula n (%)	3 (1)	4 (25)	.30 (.06–1.55)	.150
Mortality at three months n (%)	14 (24)	9 (32)	.66 (.24–1.78)	.407

Baseline demographics and comorbidities, including history of stroke and major vascular risk factors, did not differ significantly between the two craniotomy-size groups: a comparative analysis between patients dichotomized by lateral craniectomy diameter (L ≥ 6.5 cm vs. L < 6.5 cm) revealed no significant differences in those variables, as shown in Table 3.

**Table 3:** Clinical parameters, interventions, and postoperative course of patients, stratified by craniectomy size.

	Craniectomy ≥6.5 cm	Craniectomy <6.5 cm	p
Clinical parameters			
Age in years, median (IQR)	63 (58–67)	62 (55–72)	.426
Male/female, n (%)	43/17 (72/28)	21/7 (75/25)	.744
GCS at admission, median (IQR)	9 (8–11)	10 (8–12)	.795
**Comorbidities**			
Arterial hypertension, n (%)	44 (73)	19 (68)	.596
Diabetes mellitus type 2, n (%)	18 (30)	12 (43)	.236
COPD/asthma, n (%)	12 (20)	2 (14)	.125
Coronary artery disease, n (%)	5 (8)	4 (14)	.391
Atrial fibrillation, n (%)	13 (22)	6 (21)	.980
Past medical history of stroke, n (%)	6 (10)	2 (7)	.664
Prior use of oral anticoagulants, n (%)	10 (16)	5 (18)	.890
Prior use of antiplatelet agents, n (%)	16 (27)	8 (29)	.852
**Radiological parameters**			
Infarct volume, median (IQR)	54 (42–67)	48 (39–57)	.426
**Interventions**			
IV thrombolysis, n (%)	19 (34)	8 (29)	.583
Mechanical thrombectomy, n (%)	17 (31)	12 (43)	.280
Concomitant use of external ventricular drain, n (%)	46 (75)	15 (56)	.070
**Postoperative course**			
Pneumonia, n (%)	27 (45)	9 (32)	.253
Urinary tract infection, n (%)	2 (3)	3 (11)	.164

COPD – chronic obstructive pulmonary disease; GCS – Glasgow Coma Scale.

## 4 Discussion

SDC is a critical intervention for patients with SOCS, with surgical dimensions potentially influencing patient outcomes. In this cohort of 88 patients, we observed that a lateral diameter ≥6.5 cm significantly improved functional outcomes, suggesting that decompressing approximately 50 % of the cerebellar convexity may optimize surgical results. While lateral diameter emerged as a key determinant of patient recovery, other craniectomy measurements such as craniocaudal diameter and total area did not demonstrate statistically significant associations with functional improvement.

While prior case series have explored the role of SDC in cerebellar ischemic stroke and suggested optimal dimensions, none have rigorously evaluated these dimensions with comparators. In 2009, Pfefferkorn et al. (2009) analyzed 54 patients undergoing SDC for SOCS, advocating for bilateral decompression encompassing the entire posterior fossa to sufficiently relieve pressure on the cerebellum and brainstem. The authors also described resection of the posterior arch of C1 as a possible adjunct to extend craniectomy dimensions, although this decision was left to the surgeon's discretion. Consequently, no exact diameters or techniques were systematically evaluated. Similarly, Tsitsopoulos et al. conducted two studies addressing surgical treatment for bilateral (Tsitsopoulos et al., 2011a) and unilateral (Tsitsopoulos et al., 2011b) cerebellar infarctions, involving 10 and 32 patients, respectively. Both studies emphasized the importance of bilateral decompression of the posterior fossa, including the foramen magnum, and optionally the posterior arch of C1, but, like Pfefferkorn et al., did not specify exact dimensions. Conversely, Kim et al. (2016) examined the effectiveness of SDC compared to conservative treatment in a propensity-matched retrospective cohort. While this study advocated for sufficient lateral extension to reach the transverse and sigmoid sinuses, it did not establish threshold dimensions to achieve optimal outcomes.

Our study represents a pioneering effort to define precise thresholds for SDC dimensions in SOCS. Our findings suggest a critical importance of lateral extension over excessive craniocaudal expansion in improving functional outcomes. This appears to highlight that the therapeutic benefit of SDC primarily stems from adequate decompression of the cerebellar hemispheres, rather than from decompression of the foramen magnum or basal cisterns. The studies conducted by Tsitsopoulous et al. (Tsitsopoulos et al., 2011a, 2011b) also remark the importance of adequately decompressing the cerebellar hemispheres, thus adding to the body of evidence supporting an extensive lateral decompression.

Aggressive craniocaudal decompression, did not demonstrate a measurable impact on functional outcomes in our study. While we did not explicitly consider the resection of the C1 arch and foramen magnum as separate variables, these surgical measures do not appear to yield better functional results. This observation may be explained by the use of EVDs in 70 % of our cohort, which likely alleviated hydrocephalus and reduced the need for extensive cisternal decompression. Routine removal of the C1 posterior arch, as practiced in several centers, may enhance inferior decompression and facilitate tonsillar expansion. However, in our cohort, C1 removal was inconsistently performed and not evaluated as an independent variable, representing an important area for further study.

In contrast to anterior circulation ischemic strokes, where larger craniectomy sizes are associated with complications such as herniation at bone edges, hemorrhage, postsurgical hydrocephalus, and sinking skin flap syndrome (Wagner et al., 2001; Kurzbuch, 2015), our analysis did not reveal significant differences in complication rates across the dichotomized groups. Nevertheless, complications were not equally documented in all centers; hence, these results should be interpreted with caution.

Our multicentric data—collected from 11 academic institutions—underscore that the extent of suboccipital decompression remains variable and controversial, reflecting the absence of consensus regarding what constitutes an “adequate” posterior fossa decompression. To address this variability, we additionally quantified the maximum diameter of each patient's posterior fossa and calculated the ratio of the craniectomy diameter to the total posterior fossa width. This parameter allowed a more individualized assessment of decompression size, normalizing for interpatient anatomical variability. Interestingly, our results suggest that a lateral decompression encompassing approximately 50 % of the posterior fossa width—corresponding to a bone flap of about 6.5 cm—is associated with better functional outcomes. This finding supports the concept that adequate cerebellar and brainstem pressure relief can be achieved without necessarily extending the craniectomy fully to the venous sinuses, and that the relationship between decompression size and outcome is proportional rather than absolute.

### 4.1 Recommendations

Based on our results, we propose an easily applicable threshold of ≥6.5 cm lateral diameter or half of the cerebellar convexity to optimize functional outcomes while minimizing unnecessary surgical invasiveness when performing SDC in SOCS. This approach may reduce complications by preserving venous sinuses and shortening operative times. Specifically, we recommend an SDC spanning approximately 6.5 cm laterally and 4.5 cm craniocaudally on the cerebellar convexity. These dimensions, as depicted in Fig. 1, appear sufficient to achieve better functional outcomes. However, this study does not propose a theoretical redefinition of posterior fossa decompression but aims to provide empirical data describing outcome trends relative to craniectomy dimensions, acknowledging inherent anatomical and procedural variability.

### 4.2 Limitations and strengths

This study's retrospective design is a notable limitation. The decision to perform SDC was subject to institutional discretion. While we assume a relative homogeneity in medical management prior to surgery due to the academic nature of the participating institutions and their adherence to both American and European guidelines, variability in their aggressiveness in medical management cannot be completely ruled out. In this regard, osmotherapy (mannitol or hypertonic saline) was not used routinely or prophylactically at the participating centers. Because osmotherapy use was infrequent, heterogenous, and not protocolized across centers, it could not be meaningfully analyzed as a separate treatment effect in this retrospective cohort — a limitation we must explicitly acknowledge when evaluating surgical indications.

SDC also varied based on a range of clinical and surgical factors, including timing, diameter, and technique for duraplasty. Additionally, factors such as the extent of the duraplasty or the use of EVDs which may influence outcomes, were not directly assessed. The resection of the C1 arch and the foramen magnum were only indirectly assessed in the CC diameter and not as separate variables, which might have also skewed the results. Of note, we excluded patients who had primary necrosectomy in addition to SDC from our analyses. The optimum size of craniectomy in these patients remains unclear.

Approximately 10 % of patients were excluded due to incomplete imaging or unavailable functional follow-up. This exclusion was intentional to maintain a homogeneous and complete dataset for volumetric and outcome analyses. Because these exclusions were applied uniformly and without regard to treatment status or clinical trajectory, they are unlikely to introduce systematic bias, though they do reduce sample size.

Another limitation of our study relates to the use of the mRS as the primary outcome measure. Although the mRS is the most widely applied and validated scale for assessing global functional outcome after stroke—and its use facilitates comparison with prior decompressive craniectomy and ischemic stroke studies—it may not fully capture the specific neurological and functional sequelae typical of cerebellar infarction, such as ataxia, dysmetria, or balance impairment. More specialized assessment tools, such as the Barthel Index of Activities of Daily Living or the BARS (Brief Ataxia Rating Scale), may offer a more nuanced evaluation of functional recovery in this patient population. Further mRS dichotomization reflects descriptive associations rather than causal effects; however the comparability of baseline characteristics—including comorbidities and prior stroke—between groups mitigates the risk of major selection bias. Future studies should therefore aim to incorporate cerebellar-specific or multidimensional outcome scales to better characterize postoperative recovery and residual deficits following suboccipital decompressive craniectomy.

Finally, brainstem involvement is a well-established predictor of unfavorable prognosis in cerebellar infarction. Therefore, while the extent of decompression appears to influence functional recovery, our findings should be interpreted with caution, acknowledging that infarct distribution—particularly brainstem extension—represents an important confounding variable. Due to the low sensitivity of CT for detecting early brainstem ischemia and the absence of uniform pre-operative MRI across centers, an accurate and reproducible classification of brainstem involvement could not be performed. Consequently, brainstem extension remains an important unmeasured confounder, and the observed association between decompression size and functional outcome should be interpreted within this methodological context. Future prospective studies with stratification by infarct topography will be essential to isolate the independent effect of decompression size from the impact of stroke severity.

Despite these constraints, this study is the largest and only multicenter effort to date to evaluate the association between SDC dimensions and functional outcomes, providing a critical foundation for future research.

## 5 Conclusions

Our findings suggest that an SDC with a minimum lateral extension of 6.5 cm or 50 % of the posterior fossa region may improve functional outcomes at three months for patients with ischemic cerebellar stroke. Larger, prospective studies are necessary to confirm and refine these recommendations.

## Disclosure of funding

None.

## Declaration of competing interest

The authors declare that they have no known competing financial interests or personal relationships that could have appeared to influence the work reported in this paper.
